# MicroRNA Biogenesis Pathway Genes Are Deregulated in Colorectal Cancer

**DOI:** 10.3390/ijms20184460

**Published:** 2019-09-10

**Authors:** Petra Vychytilova-Faltejskova, Alena Svobodova Kovarikova, Tomas Grolich, Vladimir Prochazka, Katerina Slaba, Tana Machackova, Jana Halamkova, Marek Svoboda, Zdenek Kala, Igor Kiss, Ondrej Slaby

**Affiliations:** 1 Central European Institute of Technology, Masaryk University, Kamenice 753/5, 62500 Brno, Czech Republic; 2 Department of Surgery, University Hospital Brno, Jihlavska 340/20, 62500 Brno, Czech Republic; 3 Faculty of Medicine, Masaryk University, Kamenice 5, 62500 Brno, Czech Republic; 4 Department of Comprehensive Cancer Care, Masaryk Memorial Cancer Institute, Faculty of Medicine, Masaryk University, 62500 Brno, Czech Republic; 5 Department of Pathology, University Hospital Brno, Jihlavska 340/20, 62500 Brno, Czech Republic

**Keywords:** microRNA, biogenesis, colorectal cancer, disease-free survival, overall survival, RT-qPCR

## Abstract

MicroRNAs (miRNAs) are small non-coding RNAs that post-transcriptionally regulate gene expression. Each step of their production and maturation has to be strictly regulated, as any disruption of control mechanisms may lead to cancer. Thus, we have measured the expression of 19 genes involved in miRNAs biogenesis pathway in tumor tissues of 239 colorectal cancer (CRC) patients, 17 CRC patients with liver metastases and 239 adjacent tissues using real-time PCR. Subsequently, the expression of analyzed genes was correlated with the clinical-pathological features as well as with the survival of patients. In total, significant over-expression of all analyzed genes was observed in tumor tissues as well as in liver metastases except for LIN28A/B. Furthermore, it was shown that the deregulated levels of some of the analyzed genes significantly correlate with tumor stage, grade, location, size and lymph node positivity. Finally, high levels of DROSHA and TARBP2 were associated with shorter disease-free survival, while the over-expression of XPO5, TNRC6A and DDX17 was detected in tissues of patients with shorter overall survival and poor prognosis. Our data indicate that changed levels of miRNA biogenesis genes may contribute to origin as well as progression of CRC; thus, these molecules could serve as potential therapeutic targets.

## 1. Introduction

MicroRNAs (miRNAs) are one of the most studied subclasses of non-coding RNAs. They are short, highly conserved, single-stranded molecules 18–25 nucleotides in length that were first discovered in 1993 in *C. elegans* by Victor Ambros [1]. Since then, more than 38,500 human miRNAs have been described (www.mirbase.org, release 22.1, October 2018). Furthermore, it is estimated that these small molecules are involved in post-transcriptional regulation of more than 60% of all protein coding genes [2] due to binding to specific sites within the 3’ and 5’ untranslated regions as well as in the open reading frames of target mRNAs [3,4,5]. MiRNAs play a crucial role in many biological processes, including cell and tissue differentiation, hematopoiesis, proliferation, cell cycle regulation, stemness or cell migration [6]. Thus, their production and maturation have to be strictly regulated as any disruption of control mechanisms may lead to the development of various diseases, including cancer. 

MiRNAs sequences are distributed all throughout the genome. Initially, they were discovered in non-coding regions between genes; however, it was demonstrated that they can be derived from intergenic as well as exonic or intronic sequences [7]. The biogenesis of miRNAs starts with their transcription by RNA polymerase II (POLR2) [8] or RNA polymerase III (POLR3) [9], resulting in primary transcripts (pri-miRNAs) of several kilobases in length, which are 5’ capped and 3’ polyadenylated. The nuclear processing of pri-miRNAs into pre-miRNAs is performed by the Microprocessor complex consisting of ribonuclease (RNase) III enzyme DROSHA and its cofactor, the double-stranded RNA binding protein DiGeorge syndrome critical region 8 (DGCR8). In addition, several auxiliary factors, including the DEAD-box RNA helicases p68 (DDX5) and p72/p82 (DDX17) are important components of Microprocessor complex enabling the accuracy of DROSHA processing [10]. Subsequently, the pre-miRNA hairpins are transported to the cytoplasm via Exportin 5 (XPO5) together with Ran-GTP;3’ overhang generated by DROSHA is recognized by another RNase III enzyme DICER, which digests the pre-miRNAs into mature duplex miRNAs (18–25 nucleotides long). Importantly, the stability of DICER is increased by association with other proteins, including TAR RNA binding protein 2 (TARBP2) [11] or kinase R-activating protein (PACT) [12]. The miRNA duplex is subsequently loaded onto one of the Ago family proteins (AGO1–4), where it is unwound, and only one strand is retained in the functional miRNA-induced silencing complex (miRISC), which includes GW182/TNRC6 (trinucleotide repeat containing 6, GW182-related) proteins, GEMIN 3 (DDX20), GEMIN 4 (DDX42) and several heat shock proteins. While both strands display a knockdown potential, the “guide” strand is typically more biologically active than the “passenger” strand and this selection is influenced by several factors, including 5‘ nucleotide composition and thermodynamic properties of the duplex [13,14]. Finally, mature miRNAs guide the RISC to target sequences on mRNA transcripts to regulate gene expression via mRNA degradation or translation inhibition [15]. Although most known miRNAs follow the canonical biogenesis pathway, recent studies have reported a number of alternative ways of miRNAs maturation, including DICER-independent biogenesis [16], DROSHA/DGCR8-independent biogenesis [17], endogenous siRNA strategy [18] or small nucleolar RNA-derived miRNAs [19]. In addition, miRNAs biogenesis is regulated not only by core processing enzymes, but also by many regulatory factors, such as ADAR1/B1 (adenosine deaminase, RNA specific/B1) [20], BRCA1/2 (breast cancer genes 1/2), SMADs [21], LIN28A/B (Lin-28 homolog A/B) [22] or wild type and mutant TP53 [23] that are commonly deregulated in various cancers, including colorectal cancer (CRC). 

CRC is the third most common malignancy in both men and women worldwide and a major health problem due to its high mortality rate [24]. CRC pathogenesis is a complex process affected by various mechanisms, including both genetic and epigenetic alterations [25]. Although miRNAs have been repeatedly proven to play a critical role in the initiation and progression of this type of cancer, just a few studies have analyzed the deregulation of genes associated with miRNA biogenesis in CRC. In 2011, Faber et al. [26] first reported that over-expression of DICER is observed in patients with reduced progression free survival (PFS) as well as overall survival (OS). These results were confirmed in two independent studies [27,28] when high levels of DICER mRNA were associated with advanced clinical stage of CRC patients [28]. In addition, increased DICER expression was detected in HCT-116 cells resistant to oxaliplatin treatment [29]. Importantly, DICER impairment led to a lower expression of miR-34a, miR-126 and miR-200 family, accumulation of cancer stem cells, initiation of epithelial-to-mesenchymal transition and metastasis formation [30]. Similarly, up-regulated levels of DROSHA [27] and XPO5 [31] have been detected in CRC tissues compared to adjacent non-neoplastic tissues. While the expression of DROSHA was not associated with clinical-pathological features of patients, over-expression of XPO5 is correlated with advanced clinical stage, presence of distant metastases and worse disease-free survival (DFS) and OS, suggesting that this protein has a functional role in CRC progression [31]. Finally, Mullany et al. [32] proved that several single nucleotide polymorphisms within miRNA biogenesis genes may affect survival of CRC patients; however, the exact mechanism is unclear. 

Herein, we analyzed the expression of 19 different genes associated with miRNA biogenesis in a large cohort of CRC patients and correlated their levels with various clinical-pathological features, including survival. We have found significantly deregulated levels of 18 of these genes. In addition, the expression of some of them was elevated also in metastatic tissue, and correlation between the expression, grade, DFS and OS was observed. To our knowledge, this is the first study focused on comprehensive characterization of miRNAs biogenesis pathway deregulation in CRC.

## 2. Results

### 2.1. MiRNA Biogenesis Pathway is Significantly Deregulated in Primary Tumor and Metastatic Tissue of CRC

To evaluate the level of deregulation of miRNA biogenesis pathway in CRC, expression of 19 different genes was measured in 239 paired samples of tumor tissue and adjacent mucosa as well as in 17 samples of liver metastases by RT-qPCR. Using non-parametric Wilcoxon test for paired samples, significant over-expression of all analyzed genes was observed in tumor tissue compared to adjacent tissue (*p* < 0.0001; Table 1), except for LIN28A/B. Interestingly, while the levels of LIN28A were significantly decreased in tumor tissue (*p* < 0.0001; Table 1), its homolog LIN28B was not detected in analyzed samples. In addition, the expression of DDX5, DDX20, DGCR8, DICER1, DROSHA, EIF2C1-4, GEMIN4, TNRC6A and XPO5 was deregulated not only in tumor tissue, but also in corresponding liver metastases compared to normal mucosa (Table 1; Supplementary Figure S1). 

Subsequently, the levels of particular genes were correlated with clinical-pathological features of patients, including stage, grade, tumor location and size, lymph node positivity (LNP), and the expression of CRC biomarkers CEA (carcinoembryonic antigen) and CA19-9. It was shown that the levels of EIF2C3 and LIN28A significantly correlate with tumor stage, while the up-regulation of DDX20, DGCR8, DICER1, DROSHA, EIF2C1-2, GEMIN4, TARBP2, TNRC6A and XPO5 and down-regulation of LIN28A was observed in tumors with higher clinical grade. Furthermore, elevated levels of ADAR, DROSHA, EIF2C1/4 and TARBP2 were detected in patients with proximal tumors compared to patients with distal CRC, and expression of DICER1 and LIN28A was associated with LNP. Interestingly, the levels of 16 out of 19 analyzed genes were significantly higher in patients with tumors larger than 50 mm compared to smaller tumors. Finally, no correlation was observed between the expression of CEA, CA19-9 and analyzed genes (Table 1).

### 2.2. Deregulation of miRNA Biogenesis Pathway May Influence the Survival of CRC Patients

**Figure 1:**
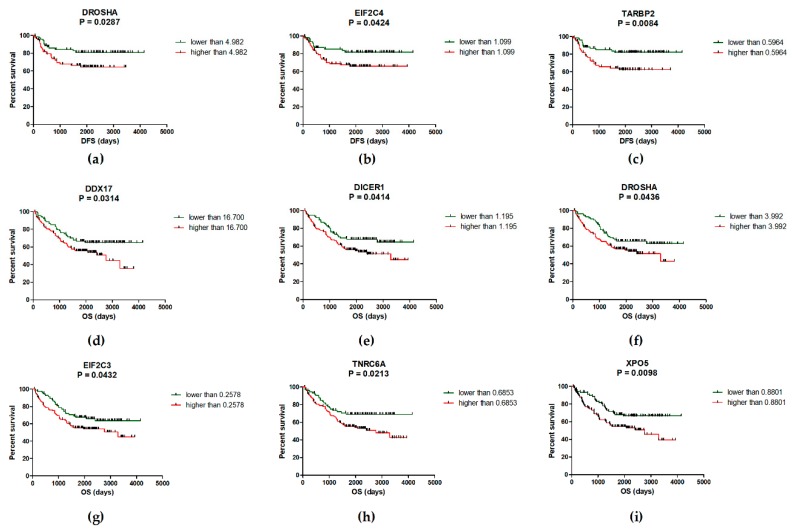
Kaplan-Meier survival analysis. High expression of DROSHA (**a**), EIF2C4 (**b**) and TARBP2 (**c**) is associated with significantly shorter disease-free survival (DFS) of CRC patients (*p* < 0.05). (d-i) Over-expression of DDX17 (**d**), DICER1 (**e**), DROSHA (**f**), EIF2C3 (**g**), TNRC6A (**h**), and XPO5 (**i**) was observed in CRC patients with significantly shorter overall survival (OS) (*p* < 0.05).

**Figure 2:**
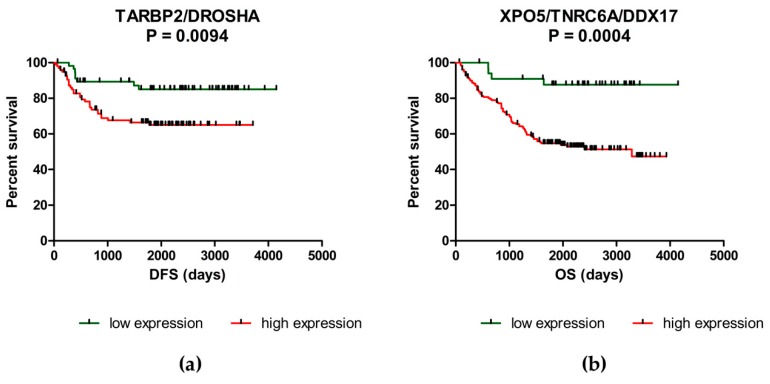
Combined Kaplan-Meier survival analysis. (**a**) The CRC patients with high expression of TARBP2 and/or DROSHA had significantly shorter DFS compared to patients with low levels of these genes (*p* = 0.0094). (**b**) The CRC patients with high levels of XPO5 and/or TNRC6A and/or DDX17 had significantly shorter OS compared to patients with low levels of these genes (*p* = 0.0004).

To assess the prognostic function of analyzed genes in CRC, Kaplan-Meier survival curves have been generated and compared by log-rank analysis. It was shown that patients with higher levels of DROSHA (*p* = 0.0287), EIF2C4 (*p* = 0.0424) and TARBP2 (*p* = 0.0084) have significantly shorter DFS compared to patients with low expression of these genes (Figure 1a–c). Concerning OS, over-expression of DDX17 (*p* = 0.0314), DICER1 (*p* = 0.0414), DROSHA (*p* = 0.0436), EIF2C3 (*p* = 0.0432), TNRC6A (*p* = 0.0213) and XPO5 (*p* = 0.0098) was associated with worse prognosis and significantly shorter OS of CRC patients (Figure 1d–i; Supplementary Table S1). Subsequently, the combined Kaplan-Meier survival analysis was performed to find the best combination of genes with prognostic function. It was found that patients with up-regulated levels of TARBP2 and/or DROSHA have significantly shorter DFS (57.5 months vs. 77.7 months; *p* = 0.0094) compared to patients with low levels of both of these genes. However, the significance was almost the same as in case of TARBP2 (*p* = 0.0094 vs. *p* = 0.0084). Concerning OS, patients with over-expression of XPO5 and/or TNRC6A and/or DDX17 had significantly shorter OS (52.1 months vs. 78.9 months) compared to patients with low levels of these genes (*p* = 0.0004) (Figure 2; Table 2). The expression levels of other genes did not correlate with DFS nor OS of CRC patients (Supplementary Table S1). 

## 3. Discussion

MiRNAs are a broad class of small non-coding RNAs 18–25 nucleotides long that play a crucial role in post-transcriptional regulation of human genes expression. They are involved in various developmental and cellular processes, including cell differentiation, proliferation, apoptosis, metabolism or migration, and their deregulation is commonly observed in many pathological conditions, including cancer [33]. Thus, their production and maturation have to be strictly regulated. The biogenesis of miRNAs is regulated on both transcriptional and post-transcriptional level and requires not only the core processing enzymes, but also many other regulatory factors, such as transcription factors or RNA binding proteins [34]. In addition, cell signaling pathways may modulate Microprocessor activity to control pri-miRNA processing, and some of the core biogenesis machinery components are subject to phosphorylation and/or acetylation [35]. However, the exact mechanisms of miRNAs biogenesis control and the effect of various protein modifications is not clear and remains to be determined. 

The objective of this study was to investigate the mRNA expression levels of 19 different genes involved in miRNA biogenesis in a large cohort of CRC patients using RT-qPCR and correlate their levels with various clinical-pathological features, including survival. We observed significant over-expression of ADAR, ADARB1, DDX5/17/20, DGCR8, DICER1, DROSHA, EIF2C1-4 (AGO1-4), GEMIN4, POLR2A, TARBP2, TNRC6A and XPO5 in tumor tissue compared to adjacent mucosa. In addition, the expression of DDX5/20, DGCR8, DICER1, DROSHA, EIF2C1-4, GEMIN4, TNRC6A and XPO5 was deregulated not only in tumor tissue, but also in corresponding liver metastases compared to adjacent tissue. Interestingly, while the levels of LIN28A were significantly decreased in tumor tissues involved in this study, its homolog LIN28B was not detected in analyzed samples. This observation is in accordance with the previous report, which describes the exclusive expression of either LIN28A or LIN28B in the majority of human colon tumors analyzed in the study [36]. Subsequently, we found a significant correlation between the expression of some of the analyzed genes and clinical stage, grade, size, tumor location and lymph node positivity. Previously, several different papers described elevated levels of DDX5 [37,38,39,40], DDX17 [38], DGCR8 [41], DICER1 [27], DROSHA [27,42], EIF2C1-4 [43,44] and XPO5 [31] in CRC. In addition, over-expression of EIF2C2-4 was associated with the presence of distant metastases [43], while the up-regulation of XPO5 correlated with the clinical stage and metastatic disease [31]. These data are in concordance with our observations. Conversely, Faggad et al. [45] observed down-regulated expression of DICER1 in tumor tissue of CRC patients and assumed that its reduced levels may contribute to disease progression. Furthermore, several different studies described up-regulation of LIN28A/B in CRC [36,46,47,48,49,50]. Finally, there are also several studies describing neither significant difference in the expression of DICER1 [28,42], DROSHA [28] and EIF2C2 [28,41] between tumor tissue and adjacent mucosa, nor in the correlation of miRNAs biogenesis genes expression with clinical-pathological features of the patients [27,41,44]. These discrepancies among the studies may be caused by numerous factors. First, the expression of analyzed genes is largely subjected to post-transcriptional modifications. Thus, mRNA levels as measured by RT-qPCR may not correlate with protein levels as detected by western blot or immunohistochemistry [51]. Furthermore, it was found that long non-coding RNAs, such as NEAT1 (nuclear enriched abundant transcript 1), are able to directly bind to the genes involved in miRNAs biogenesis and regulate their stability [52]. Importantly, recent findings suggest that most genes involved in miRNA pathway have a range of non-canonical functions and play an important role in various biological processes, such as stress response, development, transcriptional regulation or maintenance of genome integrity [53]. In addition, they are involved in diverse signaling pathways; thus, it remains controversial whether they act as tumor suppressors or oncogenes. However, it is highly probable that they might have a dual role in promoting or inhibiting tumor growth based on cancer tissue type [54,55]. 

Concerning correlation between the expression of miRNA biogenesis genes and survival, it was shown that patients with up-regulated levels of DROSHA, EIF2C4 and TARBP2 have significantly shorter DFS compared to patients with low levels of these genes. Subsequently, the over-expression of DDX17, DICER1, DROSHA, EIF2C3, TNRC6A and XPO5 was associated with worse prognosis and shorter OS of CRC patients. In addition, combined Kaplan-Meier survival analysis proved a strong prognostic potential of three-gene- panel of XPO5, TNRC6A and DDX17. Previously, higher expression of DICER1 was repeatedly detected in tumor tissue of CRC patients with poor survival [26,42]. Recently, Chen et al. [56] revealed that DICER participates in double-strand break repair by facilitating homologous recombination as well as in non-homologous end joining; thus, the overexpression of this enzyme is associated with chemoresistance. A year later, high levels of DICER were observed in CRC cells resistant to oxaliplatin and knock-down of this protein resensitized cells to this treatment [29]. In addition, several studies have shown that DICER plays a crucial role during angiogenesis and organ development [57,58]. Therefore, Vincenzi et al. [42] investigated the expression of this enzyme as prognostic and predictive factor of response to bevacizumab-based treatment in advanced CRC. Importantly, they found that low levels of DICER are observed in tissue samples of patients with better response to Bevacizumab compared to patients with high levels of this protein. Similarly, over-expression of XPO5 has been previously associated with worse clinical-pathological features and poor survival of CRC patients. In addition, the siRNA knock-down of XPO5 resulted in reduced proliferation, invasion, induction of G_1_-S cell cycle arrest and down-regulation of important oncogenic miRNAs, including miR-21, miR-10b, miR-27a, miR-182 or miR-155 in SW480 and Caco-2 cell lines [31]. These results indicate that DICER1 and XPO5 could serve as potential therapeutic targets in CRC. 

Our study has several limitations. Firstly, we did not perform global miRNA expression profiling of the studied tissue specimens. Therefore, we cannot perform correlation analysis between the deregulation of biogenetic pathway genes and miRNA expression profiles. Another limitation is the fact that we analyzed the expression of selected genes only on mRNA level. As this expression is largely subjected to post-transcriptional modifications, mRNA levels may not correlate with final protein levels. Thus, our results should be further confirmed by western blot or immunohistochemistry. Finally, further studies focusing on non-canonical functions of miRNA biogenesis factors are warranted to clarify their role in development and progression of CRC. 

In conclusion, it is well documented that miRNAs play an important role in gene regulation in both health and disease. Thus, a gradually increasing number of studies investigate the role of the central components of their biogenesis. Recently, several studies have proven that any perturbation in miRNAs biogenesis is closely associated with the development and progression of various cancers, including CRC. To our knowledge, this is the first study describing the global over-expression of 17 different genes involved in biogenesis of these small molecules, and there is a correlation of some of them with clinical-pathological features of CRC patients as well as with their prognosis. These results could be in contradiction to studies describing the general down-regulation of miRNA levels in cancer cells compared to adjacent tissues [59,60]. However, recent studies challenged this perspective, as they described a global increase instead [61,62]. In addition, accumulating evidence demonstrates the critical role of analyzed genes not only in miRNAs biogenesis, but also in expression control of many other small RNA species [63] as well as in various biological processes, including development, cellular maintenance and homeostasis [53].

## 4. Materials and Methods 

### 4.1. Patients and Tissue Samples

In total, 239 tissue samples from patients with histopathologically verified CRC (131 males, 108 females) who had undergone surgical resection at the Masaryk Memorial Cancer Institute (MMCI, Brno, Czech Republic) and University Hospital Brno (Brno, Czech Republic) from 2004–2015, as well as 239 paired adjacent non-tumoral tissues (macroscopically normal colonic epithelium) were included in the study. These tissue specimens (tumor and non-tumor) were divided into two fragments. The first fragment was stored as fresh-frozen material for gene expression analysis; the second fragment was fixed as an formal fixed and paraffin embedded (FFPE) tissue used for microscopic confirmation of cancer cell content. Only specimens with cancer cell proportion higher than 75% were included as tumor tissues, and only specimens without signs of tumor cells as non-tumor controls.

Patients’ ages ranged between 40 and 83 years with a median of 75 years. In addition, 17 samples of corresponding liver metastases from patients with metastatic CRC were used as well. All tissue samples were obtained after surgical resection and immediately placed in liquid nitrogen. All subjects enrolled in the study were of the same ethnicity (European descent) and did not receive any neo-adjuvant treatment. All patients were followed up for tumor recurrence at regular intervals, and survival time was calculated from the date of diagnosis to the date of death or last date of follow-up. Clinical and pathological characteristics including gender, age, clinical stage, grade, location, size of the tumor, CEA and CA19-9 levels were recorded and are summarized in Table 3. The concentrations of CEA and CA19-9 were measured at the Department of Laboratory Medicine at MMCI using electro-chemiluminescence immunoassay and ELECSYS® 2010 system (Roche, Basel, Switzerland); during the study, cut-offs used in clinical routine were used (CEA: 5 ng·mL^−1^; CA19-9: 27 U·mL^−1^). Written informed consent was obtained from all participants and the study was conducted in accordance with Declaration of Helsinki and approved by the local Ethics Board at MMCI.

### 4.2. Tissue Samples Preparation and Total RNA Isolation

All analyzed tissues were homogenized (MM301, Retsch GmbH, Haan, Germany) and total RNA was isolated using *mir*Vana miRNA Isolation Kit (Ambion, Austin, TX, USA; Cat. No. AM1560) according to the manufacturer’s instructions. Concentration and purity of RNA were determined spectrophotometrically by measuring its optical density (A260/280 > 2.0; A260/230 > 1.8) using a Nanodrop ND-1000 (Thermo Fisher Scientific, Waltham, MA, USA).

### 4.3. Reverse Transcription and RT-qPCR

For the purposes of gene expression analyses, cDNA was synthesized using 1000 ng of total RNA and the High-Capacity cDNA Reverse Transcription Kit (Applied Biosystems; catalog number 4368814) according to the manufacturer’s recommendations. Quantitative PCR was carried out using specific probes for selected genes listed in Supplementary Table S2 (all from Applied Biosystems) and the Applied Biosystems 7500 Sequence Detection System. 

### 4.4. Data Normalization and Statistical Analyses

The threshold cycle (Ct) data were calculated by SDS 2.0.1 software (Applied Biosystems) using the default threshold settings. All real-time PCR reactions were run in triplicate, and average threshold cycle and SD (standard deviation) values were calculated. The average expression levels of all genes in tumoral and non-tumoral tissues as well as in the metastatic tissues were normalized using PMM1 (phosphomannomutase 1) as a reference gene. This gene was chosen based on previous reports [64] and our previous experience [65]. Subsequently, all data was analyzed by the 2^-ΔCt^ method. Statistical differences between the levels of individual genes in tumor and non-tumoral tissues were evaluated by the non-parametric Wilcoxon test for paired samples. Furthermore, two-tailed Mann-Whitney *U*-test and Kruskal-Wallis test were used to analyze the correlation between the genes expression levels and clinical-pathological features of the patients. Survival analyses were performed using the log-rank test and Kaplan-Meier plots approach. All calculations were performed using GraphPad Prism version 6.00 (GraphPad Software, San Diego, CA, USA). *p*-values of less than 0.05 were considered statistically significant.

## Figures and Tables

**Table 1:** Expression levels of miRNA biogenesis genes in colorectal cancer (CRC) and their correlation with clinical-pathological features of patients.

Gene	NM ^#^ (*n* = 239)	TT ^#^ (*n* = 239)	MT ^#^ (*n* = 17)	FC (NM vs. TT) *p*-value (*n* = 239)	FC (NM vs. MT) *p*-value (*n* = 17)	Stage *p*-value	Grade *p*-value	Location *p*-value	Size *p*-value	LNP *p*-value	CEA *p*-value	CA19-9 *p*-value
ADAR	2.37 (1.43–6.78)	6.72 (3.31–18.97)	4.06 (1.64–5.65)	2.84 ***p* < 0.0001**	2.03 *p* = 0.2763	*p* = 0.2652	*p* = 0.3613	***p* = 0.0060**	***p* = 0.0004**	*p* = 0.0900	*p* = 0.1515	*p* = 0.9743
ADARB1	0.15 (0.06–0.45)	0.27 (0.12–0.78)	0.32 (0.23–0.43)	1.80 ***p* < 0.0001**	2.29 *p* = 0.0884	*p* = 0.4729	*p* = 0.1934	*p* = 0.0967	***p* = 0.0118**	*p* = 0.2357	*p* = 0.2186	*p* = 0.5291
DDX5	10.37 (5.86–19.84)	18.98 (11.35–37.92)	15.82 (10.03–27.11)	1.83 ***p* < 0.0001**	1.52 ***p* = 0.0294**	*p* = 0.8801	*p* = 0.1473	*p* = 0.0929	***p* = 0.0075**	*p* = 0.4623	*p* = 0.1383	*p* = 0.2262
DDX17	16.66 (7.85–55.75)	20.75 (11.78–75.89)	16.61 (10.99–23.14)	1.25 ***p* < 0.0001**	1.13 *p* = 0.2977	*p* = 0.3977	*p* = 0.1484	*p* = 0.0619	***p* = 0.0018**	*p* = 0.2857	*p* = 0.4826	*p* = 0.9357
DDX20	0.53 (0.30–1.27)	1.58 (0.87–4.61)	1.79 (1.38–2.26)	2.98 ***p* < 0.0001**	2.95 ***p* = 0.0092**	*p* = 0.3402	***p* = 0.0021**	*p* = 0.0520	***p* = 0.0001**	*p* = 0.5983	*p* = 0.2669	*p* = 0.1066
DGCR8	0.70 (0.42–1.28)	1.45 (0.83–2.75)	1.89 (1.38–2.24)	2.07 ***p* < 0.0001**	2.35 ***p* = 0.0261**	*p* = 0.9808	***p* = 0.0077**	*p* = 0.0900	***p* = 0.0086**	*p* = 0.1624	*p* = 0.5286	*p* = 0.2024
DICER1	1.09 (0.61–1.94)	1.70 (0.93–3.01)	2.73 (1.83–3.17)	1.56 ***p* < 0.0001**	2.34 ***p* = 0.0080**	*p* = 0.1826	***p* = 0.0177**	*p* = 0.1101	***p* = 0.0080**	***p* = 0.0075**	*p* = 0.4460	*p* = 0.6283
DROSHA	2.39 (1.27–4.19)	4.95 (2.65–9.59)	6.33 (4.86–10.01)	2.07 ***p* < 0.0001**	2.65 ***p* = 0.0033**	*p* = 0.7477	***p* = 0.0309**	*p* = 0.0212	***p* = 0.0078**	*p* = 0.1891	*p* = 0.7143	*p* = 0.2388
EIF2C1	0.63 (0.37–1.06)	1.26 (0.64–2.05)	1.72 (1.24–2.14)	2.00 ***p* < 0.0001**	2.95 ***p* = 0.0021**	*p* = 0.2571	***p* = 0.0059**	*p* = 0.0484	***p* = 0.0008**	*p* = 0.6534	*p* = 0.1351	*p* = 0.3017
EIF2C2	1.07 (0.68–1.95)	3.75 (2.57–7.33)	2.71 (2.06–4.56)	3.50 ***p* < 0.0001**	2.46 ***p* = 0.0179**	*p* = 0.7145	***p* < 0.0001**	*p* = 0.3883	***p* = 0.0422**	*p* = 0.1497	*p* = 0.7964	*p* = 0.6398
EIF2C3	0.13 (0.06–0.27)	0.31 (0.16–0.63)	0.50 (0.34–0.74)	2.38 ***p* < 0.0001**	3.58 ***p* = 0.0004**	***p* = 0.0018**	*p* = 0.0928	*p* = 0.4038	***p* = 0.0015**	*p* = 0.1236	*p* = 0.1549	*p* = 0.6630
EIF2C4	0.74 (0.45–1.38)	1.31 (0.74–2.37)	3.11 (2.44–3.92)	1.77 ***p* < 0.0001**	3.12 ***p* = 0.0004**	*p* = 0.8931	*p* = 0.0661	*p* = 0.0097	***p* = 0.0153**	*p* = 0.9047	*p* = 0.4678	*p* = 0.3493
GEMIN4	0.17 (0.10–0.29)	0.63 (0.32–1.36)	0.71 (0.47–1.03)	3.71 ***p* < 0.0001**	2.47 ***p* = 0.0045**	*p* = 0.2558	***p* = 0.0058**	*p* = 0.0656	***p* = 0.0031**	*p* = 0.2672	*p* = 0.9761	*p* = 0.7838
LIN28A	0.59 (0.09–2.38)	0.22 (0.02–1.11)	2.95 (1.30–5.40)	0.37 ***p* < 0.0001**	2.92 *p* = 0.8498	***p* = 0.0215**	***p* = 0.0140**	*p* = 0.0568	*p* = 0.8374	***p* = 0.0186**	*p* = 0.9569	*p* = 0.8718
POLR2A	1.62 (0.95–3.05)	2.54 (1.31–4.89)	1.47 (1.20–1.76)	1.57 ***p* < 0.0001**	1.37 *p* = 0.8498	*p* = 0.5469	*p* = 0.4252	*p* = 0.0642	***p* = 0.0201**	*p* = 0.2857	*p* = 0.9378	*p* = 0.2024
TARBP2	0.20 (0.12–0.39)	0.59 (0.35–1.18)	0.34 (0.29–0.47)	2.95 ***p* < 0.0001**	2.37 *p* = 0.2763	*p* = 0.3238	***p* = 0.0036**	*p* = 0.0324	***p* = 0.0044**	*p* = 0.1779	*p* = 0.5850	*p* = 0.5504
TNRC6A	0.70 (0.39–1.45)	1.08 (0.49–2.15)	1.78 (1.16–3.47)	1.54 ***p* < 0.0001**	1.81 ***p* = 0.0138**	*p* = 0.2170	***p* = 0.0021**	*p* = 0.0885	*p* = 0.0736	*p* = 0.0526	*p* = 0.3585	*p* = 0.8718
XPO5	0.23 (0.12–0.42)	1.17 (0.59–2.41)	1.33 (1.07–2.27)	5.09 ***p* < 0.0001**	7.82 ***p* = 0.0003**	*p* = 0.5897	***p* = 0.0183**	*p* = 0.8636	***p* = 0.0002**	*p* = 0.8314	*p* = 0.3712	*p* = 0.3017

NM—normal mucosa, *n*—number of samples, TT—tumor tissue, MT—metastatic tissue, FC—fold change, LNP—lymph node positivity, CEA—carcinoembryonic antigen, n. e.—non-expressed, n. a.—not applicable. ^#^ All values are given as median and 25th–75th percentile, normalized to PMM1 (2^-dCt^); bold values are statistically significant (*p* < 0.05).

**Table 2:** The detail results of combined Kaplan-Meier survival analysis.

Gene Combination	Condition	Median DFS	*p*-Value
EIF2C4/TARBP2/DROSHA ^#^	High level of 1-3 genes Low level of all three genes	58.3 months 78.5 months	0.0209
EIF2C4/TARBP2	High level of one/both genes Low level of one/both genes	58.3 months 78.5 months	0.0343
EIF2C4/DROSHA	High level of one/both genes Low level of one/both genes	57.5 months 78.5 months	0.0224
TARBP2/DROSHA	High level of one/both genes Low level of one/both genes	57.5 months 77.7 months	0.0094
**Gene Combination**	**Condition**	**Median OS**	***p*-Value**
XPO5/TNRC6A/DDX17/ DICER1/EIF2C3/DROSHA *	High level of 3–6 genes Low level of 4–6 genes	51.5 months 73.6 months	0.0020
XPO5/TNRC6A	High level of one/both genes Low level of one/both genes	52.3 months 71.1 months	0.0024
XPO5/TNRC6A/DDX17	High level of 1–3 genes Low level of all three genes	52.1 months 78.9 months	0.0004

^#^ High level of EIF2C4 (DFS) = normalized expression higher than 1.0990; high level of TARBP2 (DFS) = normalized expression higher than 0.5964; high level of DROSHA (DFS) = normalized expression higher than 4.9820. * High level of XPO5 (OS) = normalized expression higher than 0.8801; high level of TNRC6A (OS) = normalized expression higher than 0.6853; high level of DDX17 (OS) = normalized expression higher than 16.7000; high level of DICER1 (OS) = normalized expression higher than 1.1950; high level of EIF2C3 (OS) = normalized expression higher than 0.2578; high level of DROSHA (OS) = normalized expression higher than 3.9920.

**Table 3:** Clinical-pathological characteristics of study subjects.

Characteristics	Number of Patients (*n*)
**Number**	*n* = 239
Age (mean ± SD) *, years	75 ± 11
**Sex, number (%)**	
Male	131 (55)
Female	108 (45)
**TNM stage, number (%)**	
Stage I	41 (17)
Stage II	77 (32)
Stage III	55 (23)
Stage IV	66 (28)
**Grade, number (%)**	
Grade 1	64 (27)
Grade 2	119 (50)
Grade 3	52 (22)
Unknown	4 (1)
**Location, number (%)**	
Distal	142 (59)
Proximal	96 (40)
Unknown	1 (1)
**Tumor size, number (%)**	
< 50 mm	157 (66)
≥ 50 mm	54 (23)
Unknown	28 (11)
**Pre-CEA levels ^†^, number (%)**	
< 5 ng · mL ^−1^	70 (29)
≥ 5 ng · mL ^−1^	68 (29)
Unknown	101 (42)
**Pre-CA19-9 levels ^‡^, number (%)**	
< 27 U · mL ^−1^	110 (46)
≥ 27 U · mL ^−1^	28 (12)
Unknown	101 (42)

* SD—standard deviation, ^†^ pre-CEA—preoperative levels of carcinoembryonic antigen, ^‡^ pre-CA19-9—preoperative levels of CA19-9.

## Supplementary Materials

Supplementary materials can be found at https://www.mdpi.com/1422-0067/20/18/4460/s1.

## Abbreviations

miRNAs	microRNAs
POLR2/3	RNA polymerase II/III
pri-miRNAs	primary microRNAs
RNase	ribonuclease
DGCR8	DiGeorge syndrome critical region 8
XPO5	exportin 5
TARBP2	TAR RNA binding protein 2
PACT	kinase R-activating protein
miRISC	miRNA-induced silencing complex
TNRC6	trinucleotide repeat containing 6
ADAR1/B1	adenosine deaminase, RNA specific/B1
BRCA1/2	breast cancer genes 1/2
LIN28A/B	Lin-28 homolog A/B
CRC	colorectal cancer
OS	overall survival
DFS	disease-free survival
LNP	lymph node positivity
CEA	carcinoembryonic antigen
NM	normal mucosa
n	number of patients
TT	tumor tissue
MT	metastatic tissue
FC	fold change
n. e.	non-expressed
n. a.	not applicable
NEAT1	nuclear enriched abundant transcript 1
SD	standard deviation
PMM1	phosphomannomutase 1
