# Supplementary material for: MicroRNA Biogenesis Pathway Genes Are Deregulated in Colorectal Cancer
Source: Int J Mol Sci. 2019 Sep 10;20(18):4460. doi: 10.3390/ijms20184460 (PMC6770105; doi:10.3390/ijms20184460)
Supplement: Supplementary file 1 [file ijms-20-04460-s001.zip › Supplementary Figure 1.docx]

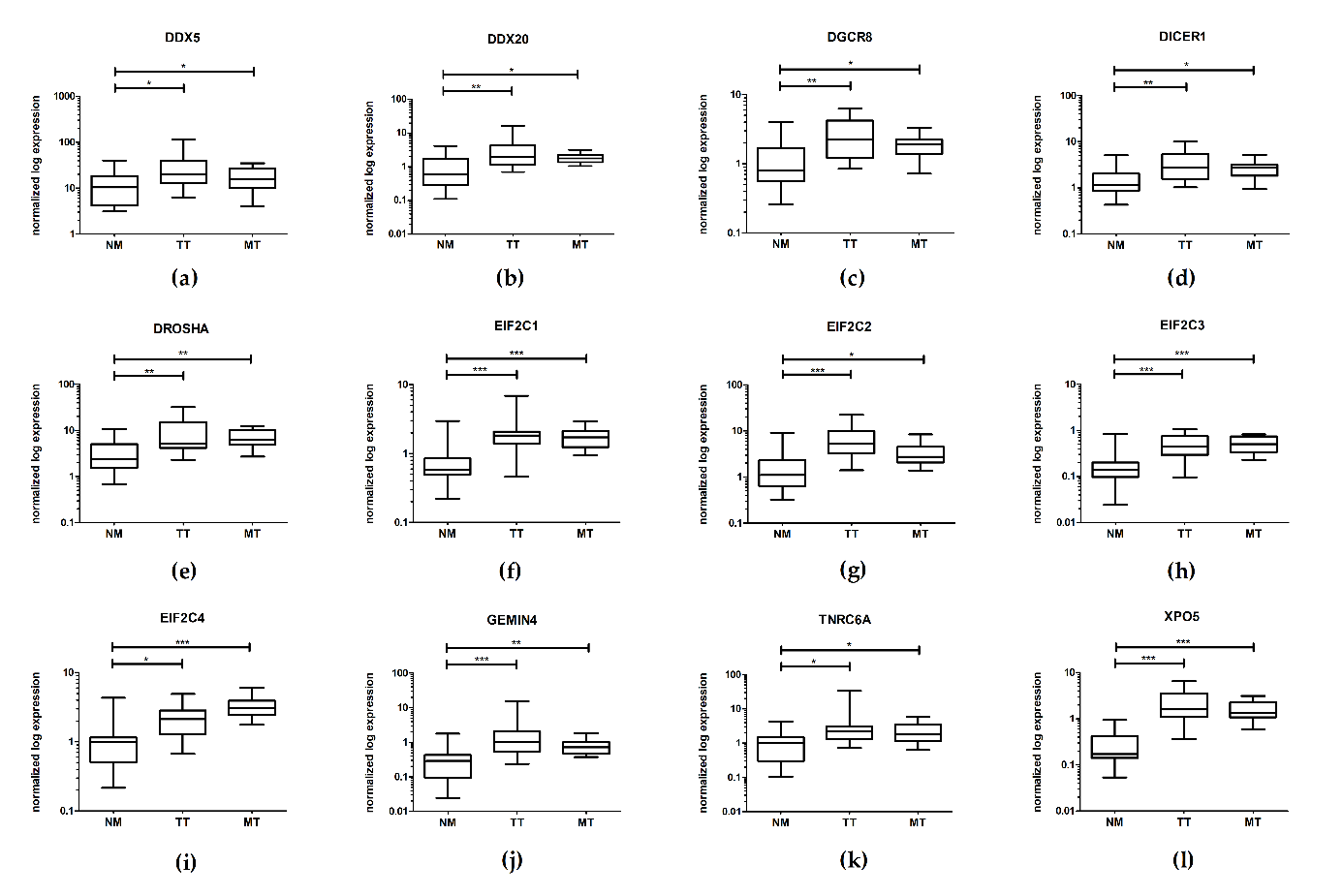


**Supplementary Figure 1: Deregulated expression of miRNA biogenesis genes in primary tumor tissue and liver metastases.** (a-l) Expression of DDX5, DDX20, DGCR8, DICER1, DROSHA, EIF2C1-4, GEMIN4, TNRC6A, and XPO5 is significantly up-regulated not only in primary tumor tissue but also in liver metastases of CRC patients compared to adjacent mucosa. *P < 0.05; **P < 0.01; ***P < 0.001
