# Supplementary material for: MicroRNA Biogenesis Pathway Genes Are Deregulated in Colorectal Cancer
Source: Int J Mol Sci. 2019 Sep 10;20(18):4460. doi: 10.3390/ijms20184460 (PMC6770105; doi:10.3390/ijms20184460)
Supplement: Supplementary file 1 [file ijms-20-04460-s001.zip › Supplementary Table 1.docx]

**Supplementary Table 1:** Prognostic potential of genes involved in miRNAs’ biogenesis.

|  | **DFS** | | **OS** | |
| --- | --- | --- | --- | --- |
| **Gene** | **cut-off value^#^** | **P-value** | **cut-off value^#^** | **P-value** |
| ADAR | 4.4420 | 0.7806 | 4.1270 | 0.8186 |
| ADARB1 | 0.2261 | 0.3440 | 0.1934 | 0.2022 |
| DDX5 | 17.530 | 0.2587 | 14.870 | 0.1785 |
| DDX17 | 20.730 | 0.1741 | 16.700 | **0.0314** |
| DDX20 | 1.1250 | 0.9315 | 1.1370 | 0.2547 |
| DGCR8 | 1.2970 | 0.5192 | 1.0060 | 0.9677 |
| DICER1 | 1.3660 | 0.5215 | 1.1950 | **0.0414** |
| DROSHA | 4.9820 | **0.0287** | 3.9920 | **0.0436** |
| EIF2C1 | 0.9125 | 0.4095 | 0.9124 | 0.1180 |
| EIF2C2 | 3.4790 | 0.2185 | 3.2140 | 0.3347 |
| EIF2C3 | 0.2091 | 0.9264 | 0.2578 | **0.0432** |
| EIF2C4 | 1.0990 | **0.0424** | 0.9910 | 0.5172 |
| GEMIN4 | 0.4730 | 0.2215 | 0.4809 | 0.2613 |
| LIN28A | 0.0669 | 0.6329 | 0.0653 | 0.1038 |
| POLR2A | 2.1670 | 0.2447 | 3.9630 | 0.8511 |
| TARBP2 | 0.5964 | **0.0084** | 0.4344 | 0.7243 |
| TNRC6A | 0.6793 | 0.3645 | 0.6853 | **0.0213** |
| XPO5 | 0.9369 | 0.3998 | 0.8801 | **0.0098** |

DFS – disease-free survival; OS – overall survival; ^#^expressed as 2^-dCt^; bold values are statistically significant (P < 0.05)
