# Supplementary material for: MicroRNA Biogenesis Pathway Genes Are Deregulated in Colorectal Cancer
Source: Int J Mol Sci. 2019 Sep 10;20(18):4460. doi: 10.3390/ijms20184460 (PMC6770105; doi:10.3390/ijms20184460)
Supplement: Supplementary file 1 [file ijms-20-04460-s001.zip › Supplementary Table 2.docx]

**Supplementary Table 2:** List of analyzed genes and assays ID (all from Applied Biosystems).

| **Gene abbreviation** | **Name of gene** | **Assay ID** |
| --- | --- | --- |
| ADAR | Adenosine deaminase, RNA specific | Hs00241666_m1 |
| ADARB1 | Adenosine deaminase, RNA specific B1 | Hs00953724_m1 |
| DDX5 | DEAD-box helicase 5 | Hs00189323_m1 |
| DDX17 | DEAD-box helicase 17 | Hs00428757_m1 |
| DDX20 | DEAD-box helicase 20 | Hs00200516_m1 |
| DGCR8 | DiGeorge syndrome critical region 8 | Hs00256062_m1 |
| DICER1 | Dicer 1, ribonuclease III | Hs00229023_m1 |
| DROSHA | Drosha, ribonuclease III | Hs00203008_m1 |
| EIF2C1 | Eukaryotic translation initiation factor 2C, 1 | Hs01084653_m1 |
| EIF2C2 | Eukaryotic translation initiation factor 2C, 2 | Hs01085579_m1 |
| EIF2C3 | Eukaryotic translation initiation factor 2C, 3 | Hs01087121_m1 |
| EIF2C4 | Eukaryotic translation initiation factor 2C, 4 | Hs01059731_m1 |
| GEMIN4 | Gem nuclear organelle associated protein 4 | Hs00249038_m1 |
| LIN28A | Lin-28 homolog A | Hs00702808_s1 |
| LIN28B | Lin-28 homolog B | Hs01013729_m1 |
| PMM1 | Phosphomannomutase 1 | Hs00160195_m1 |
| POLR2A | RNA polymerase II subunit A | Hs00172187_m1 |
| TARBP2 | TARBP2, RISC loading complex RNA binding subunit | Hs00366328_m1 |
| TNRC6A | Trinucleotide repeat containing 6A | Hs00325721_m1 |
| XPO5 | Exportin 5 | Hs00382453_m1 |
